# The Complex and Diverse Genetic Architecture of the Absence of Horns (Polledness) in Domestic Ruminants, including Goats and Sheep

**DOI:** 10.3390/genes13050832

**Published:** 2022-05-06

**Authors:** Rebecca Simon, Cord Drögemüller, Gesine Lühken

**Affiliations:** 1 Institute for Animal Breeding and Genetics, Justus Liebig University Giessen, 35390 Giessen, Germany; rebecca.simon@agrar.uni-giessen.de (R.S.); gesine.luehken@agrar.uni-giessen.de (G.L.); 2 Institute of Genetics, Vetsuisse Faculty, University of Bern, 3012 Bern, Switzerland

**Keywords:** horn development, hornless, intersexuality, Bovidae, bovine, caprine, ovine, ruminants, genome editing

## Abstract

Horns are the most obvious common feature of Bovidae. The naturally occurring absence of horns in these species, also known as polledness, is of surprisingly heterogeneous nature, although they are Mendelian traits. This review compares in detail the molecular differences among the causes of inherited polledness in the domestic ruminant species of cattle, yak, sheep, and goat based on the causal gene variants that have been discovered in recent years. The genetic causes for the lack of horns in small ruminants seem not only to be more complex, e.g., in sheep, breed-specific characteristics are still unexplained, but in goats, there is also the associated disorder of intersexuality—polled intersex syndrome (PIS). In connection with animal welfare and the associated discussion about a legal ban on the dehorning of all farm animals, naturally hornless animals and the causal genetic variants are of increasing research interest in the age of genome editing. However, the low acceptance of genetic engineering in livestock, especially in European societies, limits its use in food-producing animals. Therefore, genotype-based targeted selection of naturally occurring variants is still a widely used method for spreading this desired trait within and across populations, at least in cattle and sheep.

## 1. Horns in Bovid Species

During evolution, pecorans (i.e., higher ruminants) developed a notable diversity of bony skull attachments called "headgear", which are likely to have an identical genetic origin [1]. Ruminants are the only living group of mammals that have bony (osseous) headgear that is covered by a non-deciduous, unforked keratinous sheath [2]. Recently, comparative transcriptome analyses showed that bovine horns and cervid antlers share similar gene expression profiles and a common cellular basis that develops from neural crest stem cells [2]. Polyceraty, the presence of more than two horns, known in sheep and goat and observed since ~6000 BCE, is associated with defective HOXD1 function due to natural mutations [1].

**Figure 1:**
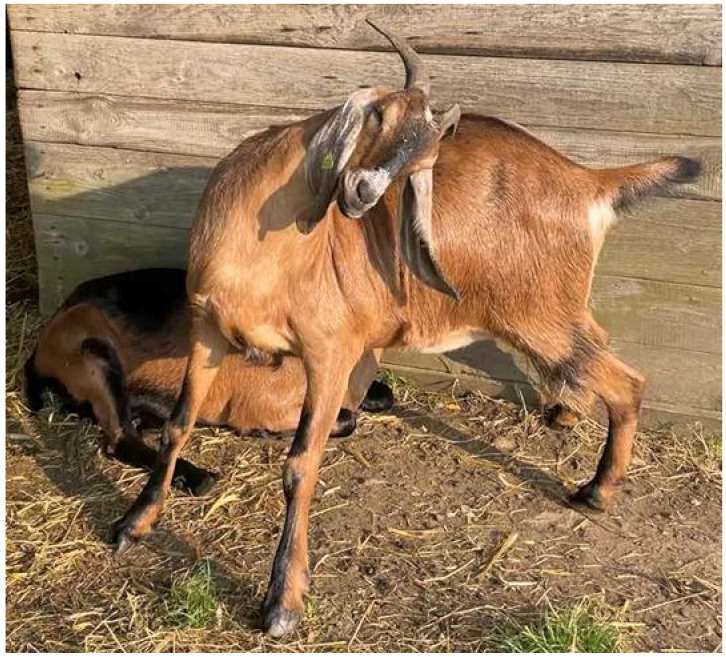
Anglo Nubian goat scratching its back with the horn tip.

Horns in bovids, the biological family of cloven-hoofed, ruminant mammals, including cattle, goat, and sheep, play a role in social behavior and protection. It is assumed that horns serve as a weapon in self-defense against predators and in ranking fights, for example, by stabilizing the head position during fights, as well as in sexual selection through intramale competition. Even impact absorption by horns is possible [3,4,5,6,7]. Horns can also be an attribute to the animal’s social status and play a crucial role in mating success [8]. It has been shown that horns offer comparable advantages for females, as they do for males, in the competition for resources [9]. Furthermore, depending on their shape and size, horns can be used as tools in body care (Figure 1). In so-called “biodynamic agriculture”, it is thought that the horns are important for the animal’s thermoregulation or digestive processes [10,11].

However, naturally occurring, genetically hornless (polled) animals are more or less common in most domesticated Bovidae, including various important livestock species (e.g., cattle, buffalo, yak, sheep, and goat) [12]. In general, hornlessness is apparently not associated with serious health restrictions. The reported fertility restrictions in hornless goats are an exception and will be discussed later. In addition, Stookey and Goonewardene (1995) showed that the polled condition in beef cattle bulls on performance testing stations had no disadvantages in the analyzed performance parameters compared to horned animals [13]. Furthermore, there is no evidence of a pleiotropic effect of the polled trait on the milk yield, fat content, somatic cell count as an indicator of mastitis, or female fertility in cows [14]. In the feral Soay sheep population on St. Kilda island, it has even been observed that horned rams have a higher annual breeding success but a shorter expectancy than scurred males, which have loosely attached horns with no bony connection [15].

## 2. Impact of Horn Status on the Welfare of Humans and Animals

**Figure 2:**
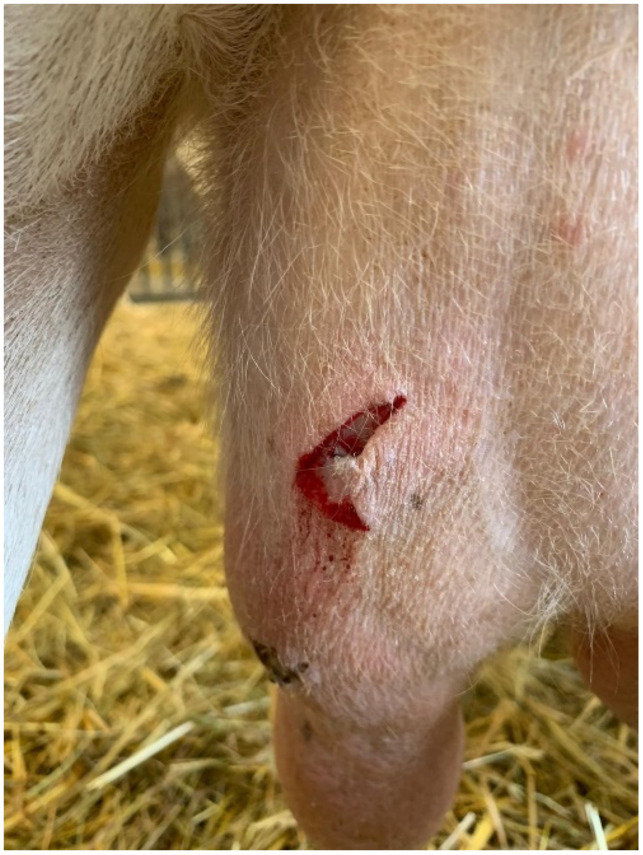
Fresh (in focus) and older, already crusty injury (at the base of the teat) on the left udder of a Saanen goat. The injuries were caused by horn blows from horned flock mates.

Horned animals pose a danger when interacting with humans [16] and flock mates [17,18]. Bruising, which reduces meat quality [19], or serious injuries to the udder (Figure 2), which decreases the milking yield, can be the result of attacks on animals by horned individuals. The risks can be reduced, if possible, by adjusting the housing management [20,21] but cannot be eliminated completely. Therefore, many farmers prefer polled animals [22]. The disbudding of horned calves and goat kids is a painful standard husbandry procedure to reduce the described risks of injuries [22,23]. To address animal welfare concerns, the often-performed practice of dehorning is regulated by law in many countries. The European Council Directive 98/58/EC (last updated 2019), which states the minimum standards for the protection of farm animals, is the basis for the regulation of dehorning in the European Union [24]. EU member states have their own national agreements on the dehorning of livestock, which vary widely in stringency [25]. For example, in Germany, the Protection of Animals Act (Tierschutzgesetz, TierSchG) regularizes interventions on animals [26]. The physical removal of horns is generally prohibited unless there is a veterinary indication in an individual case (§6 TierSchG). There is an exception for calves younger than six weeks: dehorning without anesthesia is still allowed (§5 TierSchG), even if this condition is already in the focus of discussion and an animal-friendly alternative is demanded [27]. For organic farming, the EU legislation prohibits dehorning as a routine treatment, but local authorities can authorize exceptions [28]. Even though dehorning is partly legal, it is associated with suffering and pain for the animal [17,29]. Therefore, the need for and interest in genetically polled animals is increasingly apparent.

## 3. Diversity of Horn Status in Domestic Ruminants

There is a wide variety of naturally occurring forms of horn size, shape, and position, including rare forms of hornlessness (polledness) [30,31,32,33]. Breeds can have only one characteristic horn phenotype, i.e., be fully horned, such as Highland cattle and German Grey Heath sheep, or be hornless without exception, such as Aberdeen Angus cattle [12]. In general, the polled trait is more common in beef cattle than in dairy breeds. This man-made, breeding-induced differentiation can be explained by differences in animal husbandry and handling (e.g., temporary or permanent fixation vs. free-range with little or no restraint) [34]. In dairy cattle, for example, daily fixation and human contact during the lactation period are common. The reason that, in contrast to cattle and sheep, no completely polled goat breeds are known so far, will be explained later. On the other hand, there are numerous breeds in which the horn status varies, i.e., in both sexes polled and horned animals occur (e.g., Charolais cattle, Holstein Friesian cattle [12]). There are also sex-linked horns, which are the most common in many sheep breeds (e.g., Romanov sheep [12]).

In cattle and sheep, another form of horn growth is known: scurs. Scurs are hornlike formations that occur occasionally in a wide variety of sizes and forms as an unexpected phenotype when breeding polled cattle or sheep [35,36]. These appendages are smaller, deformed, and not as firmly attached to the skull as normal horns [37]. In goats, the scurs phenomenon has not been proven, but breeders sometimes report similar horn-like structures, such as wiggle horns.

## 4. Molecular Causes of Inherited Absence of Horns in Domestic Ruminants

The development of horns involves hundreds of genes [2]. Since patterning and differentiation of horn precursor cells occurs early during embryogenesis, it is experimentally difficult or almost impossible to study [38]. Therefore, natural mutations affecting horn growth, such as polledness, offer a valuable alternative for studying the underlying molecular and cellular mechanisms. Numerous studies have shown that the genetic causes of polledness are different in cattle (OMIA 000483-9913), yak (OMIA 000483-30521), sheep (OMIA 000483-9940), and goats (OMIA 000483-9925). The heterogeneity now known suggests that the corresponding mutations affecting different genes occurred independently of each other in the different species. Therefore, the current state of the knowledge on the molecular genetic causes for polledness is described below for each species individually. In particular, it is shown how different the genetic backgrounds are in sheep and goats compared to bovines such as cattle and yak.

### 4.1. Cattle (Bos taurus and Bos indicus) and Mongolian Yak (Bos mutus)

**Figure 3:**
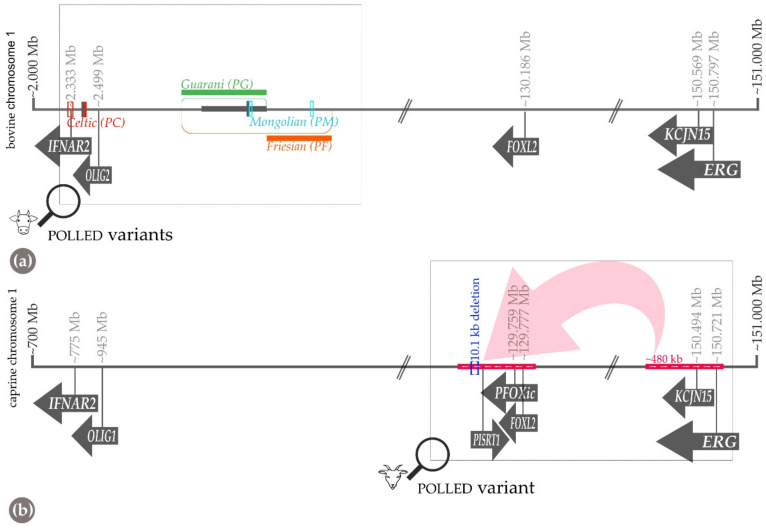
Schematic representation of the location of the different known polledness-causing variants on bovine (**a**) and caprine (**b**) chromosome 1. Note the corresponding genes annotated in both species have been drawn one below the other for simplicity but are in different positions on the respective chromosome. The coordinates refer to the current reference genome of cattle (ARS-UCD 1.2) and goat (ARS1).

Polledness in the cattle population has been a known trait for millennia. Hornless dairy cows were already depicted in ancient Egyptian artwork, such as on the sarcophagus of Queen Kawit [39]. The earliest findings of polled cattle in Germany are dated to 4000–6000 years BCE, about 2500 years after the first evidence of domestication [34]. Schafberg and Swalve reviewed that since the 20th century, the occurrence of polled cattle along with the breeding has increased slightly, but is still not in the focus of most developed breeding programs [34]. A recently published review gives a comprehensive overview of the different aspects of inherited polledness in cattle [40]. The *POLLED* locus (*P*) in cattle is located on bovine chromosome 1 [41]. Polledness is inherited as an autosomal monogenic dominant trait, with allele *P* (hornless) dominating allele *p* (horned) [42,43]. Two different *P* alleles can occur in hornless cattle depending on their origin: the “*Celtic*” polled allele (*PC* or *P_202ID_*) of Scandinavian and British origin and the “*Friesian*” allele (*PF* or *P_80kbID_*), which occurs in cattle of Holstein Friesian origin. Rarely, there are also compound heterozygous animals (e.g., in polled Simmental cattle by crossing with hornless Red Holsteins) [44]. The causal variant of the *PF* allele represents a tandem duplication of an 80-kb segment (Figure 3) [45]. In contrast, the *PC* allele is a smaller-sized complex insertion/deletion variant with a duplication of 208 bp, inserted after 10 bp of the wildtype sequence in combination with a 6-bp deletion [44,46]. Both bovine variants do not affect protein-coding genes but most likely alter the expression of the noncoding RNAs that are relevant for horn bud formation during early embryonal development. Mariasegaram et al. (2010) described the gene networks involved in the development of horns and scurs but did not find differentially expressed genes involved that map to the *P* locus on bovine chromosome 1 [47]. However, a subsequent RT-PCR-based expression analysis revealed the importance of *relaxin/insulin-like family peptide receptor 2* (*RXFP2*) and *forkhead box L2* (*FOXL2*) expression for horn development, as an overexpression was observed in the horn bud area [48]. Interestingly, as detailed later, these two genes are associated with horn growth in sheep and goat, respectively [49,50].

In wild yaks (*Bos mutus*), a third “*Mongolian*” allele (*PM* or *P219bpID*) for polledness, affecting the same genomic locus on chromosome 1, was discovered [51]. It is a complex 219-bp duplication/insertion in combination with a 7-bp deletion and 6-bp insertion located 621 bp upstream, resulting in a duplication of an 11-bp motif that is entirely conserved among Bovidae [51] (Figure 3).

The fourth currently known bovine *POLLED* allele, designated as *PG*, was found in polled Nellore cattle (*Bos indicus*). This variant is a 110-kb tandem duplication located in the same genomic region on bovine chromosome 1 (Figure 3) [52]. 

With the knowledge of the variants explaining the different *POLLED* alleles in cattle, genetic testing is possible, although genotyping of the structural variants, in particular, can be challenging. A detailed comparison of different methods was recently published [53].

Little is known about the genetic background of horns or potential hornlessness in further bovids. For example, in water buffalo (*Bubalus bubalis*), genetically polled individuals are known to occur, but the underlying variant(s) remain unknown (OMIA 000483-89462). 

In addition to horned and polled cattle, there are also some with an intermediate phenotype, the so-called scurs or “wiggle horns”. Animals showing scurs are heterozygous for one of the polled alleles [48]. The development of scurs in cattle cannot be explained by a single locus, as GWAS studies did not show clear results [36]. The original and still widely accepted model for the inheritance of horns and scurs [42] has recently been rejected. Presumably, an oligogenic model explains the development of scurs in cattle. Capitan (2011) stated that additionally to the type I scurs mentioned by Asai (2004), a quite similar but independent form of scurs (type II) that does not segregate for a known *POLLED* allele was noticed in a single Charolais cattle family. A causative frame-shift variant in the *twist family bHLH transcription factor 1* (*TWIST1*) gene on bovine chromosome 4, representing a loss-of-function allele, was found and highlights the genetic complexity of horn-growth phenotypes in cattle [54].

### 4.2. Goat (Capra hircus)

**Figure 4:**
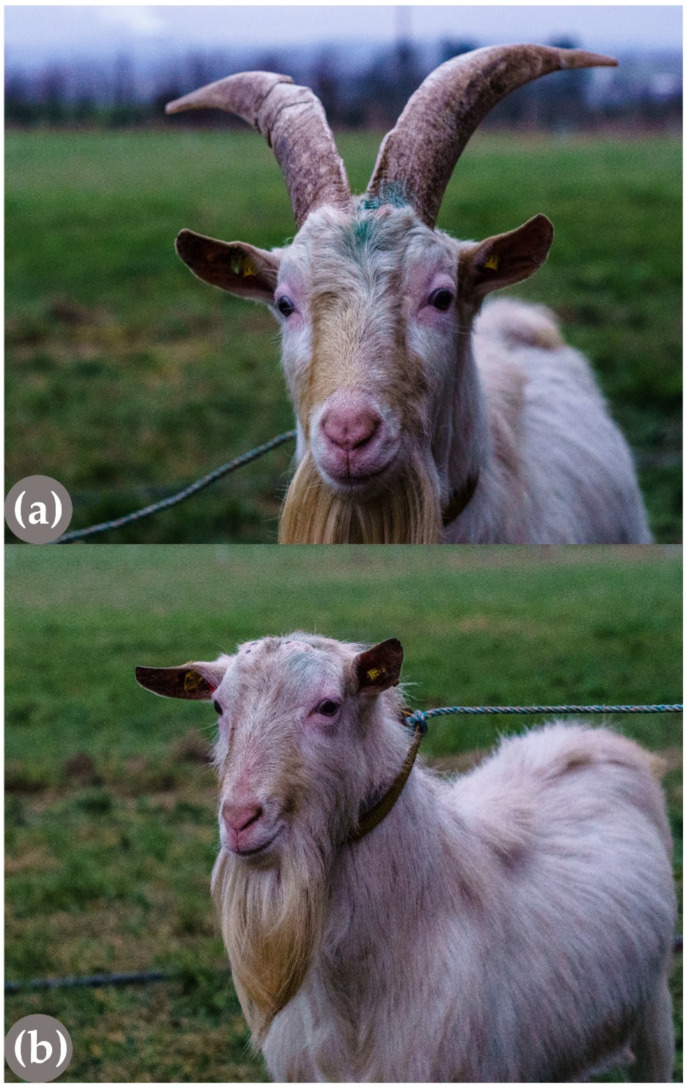
A horned (**a**) and heterozygous polled (**b**) Saanen buck, sired by the same heterozygous polled father. Both rams were about 1.5 years old when the pictures were taken (C. Barth).

As in cattle, polledness in goats (Figure 4) follows a monogenic autosomal dominant mode of inheritance. A complex structural genetic variant characterized by the fusion of a large 480-kb-sized duplicated chromosome 1 segment into the previously reported deleted part of 10 kb further upstream on chromosome 1 [55] causes the absence of horn growth in goats [56] (Figure 3). Recently, the presence of this complex structural variant was also confirmed in Chinese goat breeds with polled animals [57]. 

**Figure 5:**
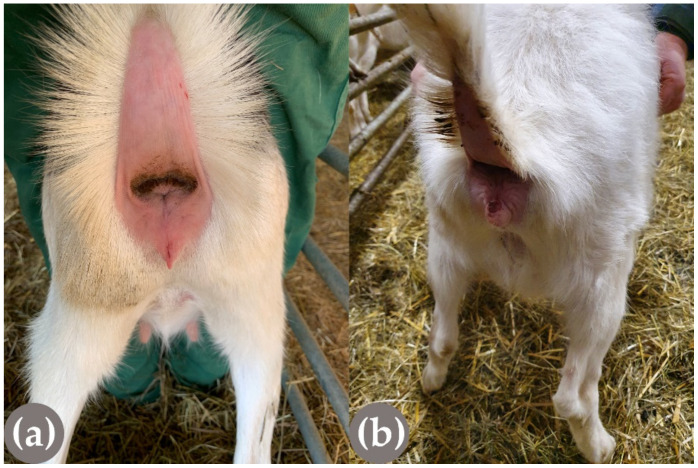
Comparison of the external genitalia of a normal (**a**) and PIS-affected (**b**) female (XX) homozygous polled Saanen goat.

So far, it is unclear which elements of these complex structural variants contribute to the lack of horn growth in goats. In contrast to what is known from other species, the dominantly inherited polledness in goats is associated with recessive intersexuality [58] (Table 1). The so-called polled intersexuality syndrome (PIS) was first observed in polled flocks by an abnormal sex ratio, i.e., a higher-than-average number of phenotypic males [59,60]. Homozygous polled females (60, XX) are infertile intersexes. They show a variable phenotype ranging from “normal” female to “normal” male, including all possible combinations in between (Figure 5). Therefore, it is difficult to identify some of these hornless intersexual animals as such [61]. Generally, intersexuality is not observed in genetically male (XY) homozygous polled goats. However, there is non-scientific evidence that hornlessness can also be associated with fertility problems in bucks. This is currently being investigated. Due to the known inheritance, it is possible to the avoid excessive appearance of PIS-affected offspring by well-planned breeding in goats (e.g., [62]), but the establishment of a fully polled and fertile flock is still impossible. Due to these new findings on the molecular background of polledness in goats, genetic testing for PIS is now possible as well. With genotyped animals, planned breeding is facilitated and polled intersexes with an inconspicuous phenotype of the genitalia can already be identified early in life [56,57].

Functionally, the originally reported PIS-causing 11-kb deletion published by Pailhoux et al. (2001) affects elements that regulate the transcription of *FOXL2*, which is also implemented in the polledness of cattle as mentioned above [55,63]. Obviously, homozygosity for the deletion leads to a decreased transcription of these genes in the goat’s ovaries [55]. The recently described refined breakpoints in that region of goat chromosome 1 are located in the *FOXL2* topologically associating domain (TAD) when compared to the corresponding human genome region, i.e., in the regulatory domain responsible for *FOXL2*. Duplications of genomic regions are associated with various disorders, but the phenotypes, which are thought to arise from an increase in gene copy number, often cannot be explained by changes in gene dosage [64]. However, genomic duplications that change the structure and function of topologically associated domains (TADs) can cause phenotypes without altering the gene copy number [65]. TADs are chromosomal regions with an increased frequency of internal chromatin interactions, e.g., between genes and their distal regulatory elements.

In humans, the duplication of a region between *SRY-box transcription factor 9* (*SOX9*) and *potassium inwardly rectifying channel subfamily J member 2* (*KCNJ2*) that lies within the *SOX9* TAD results in sex reversal from female to male. In contrast, an inter-TAD duplication that involves sex reversal duplication and spans into the *KCNJ* TAD—but without the *KCNJ* genes included—has no influence on the phenotype [65]. In goats, the duplicated 480 kb-sized genomic segment of the PIS-associated variant contains the *KCNJ* gene and parts of the *ETS transcription factor ERG* (*ERG*) gene as well as parts of the respective TADs. Therefore, the duplicated segment contains a boundary that separates the TADs and, thus, the regulatory domains from each other. When the duplicated segment is inserted into the breakpoint of the *FOXL2* region, it can be assumed that a fusion TAD (neo-TAD) is formed, consisting of one part of the duplication and the remaining of the *FOXL2* TAD. Due to the inversion, *KCNJ* is placed on the other side of the boundary and is, therefore, isolated (Figure 3). Therefore, it could be speculated that the "residual" of the *ERG* gene is of functional importance. This part also contains enhancers and could, therefore, lead to ectopic expression of *FOXL2* in developing horn buds. Future research might evaluate the hypothesis that the caprine PIS variant represents a loss-of-function of *FOXL2*, as parts of the regulatory domain are missing, leading to ectopic expression in addition to the presence of a gain-of-function through the *ERG* enhancers.

### 4.3. Sheep (Ovis aries)

**Figure 6:**
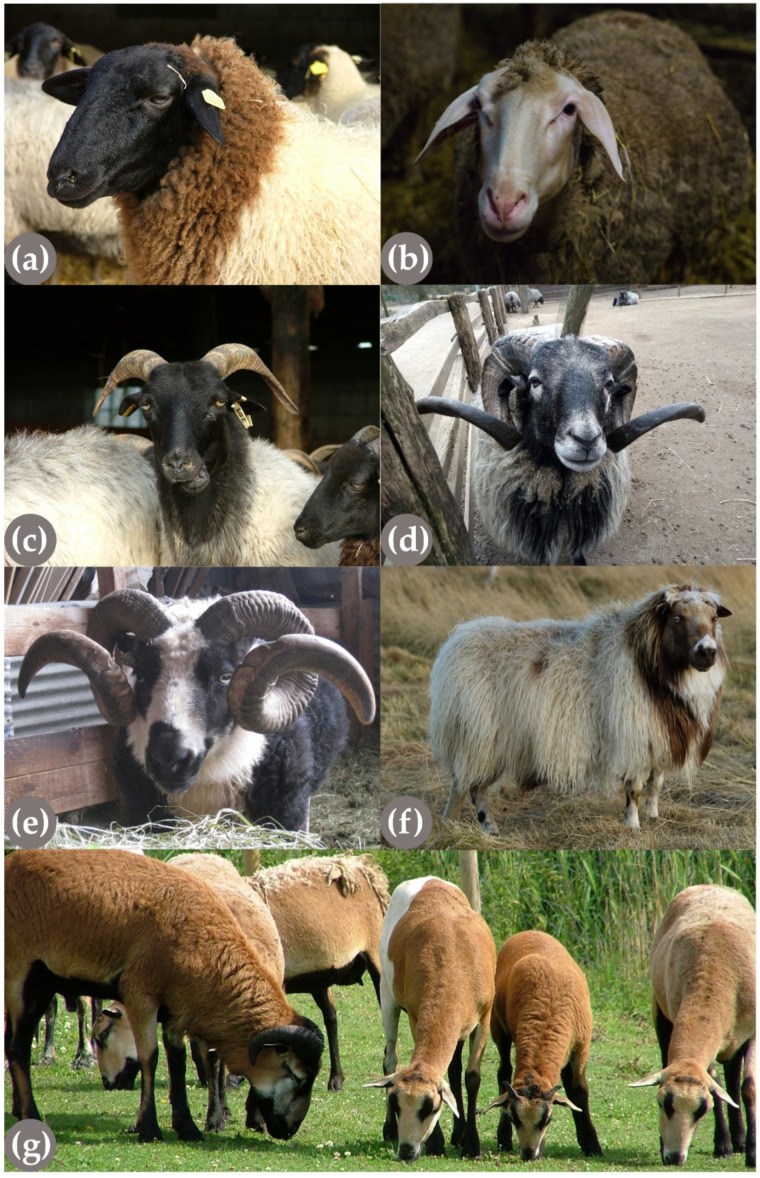
Sheep breeds (*Ovis aries*) showing different horn phenotypes belonging to different horn groups. Rhone Sheep (**a**) and Merinoland Sheep (**b**) (C. Barth) are typical representatives of the group of completely polled breeds. On the contrary, in breeds in which horns are fixed, both individuals develop horns, exemplified by a ewe (**c**) and a ram (**d**) of the German Grey Heath sheep breed. As an example of a breed in which horn status is variable in both sexes, two Icelandic sheep rams, horned (**e**) and hornless (**f**), are shown (K. Elísabetardóttir). In other breeds, the horn status is linked to the sex. For example, in the Cameroon sheep (**g**), the rams are always horned (ram on the left side and male lamb in the middle) and the females are always hornless (ewe in the middle and on the left side of the picture). Please note that a list of sheep breeds belonging to different horn status groups can be found in Table 2.

Polledness in sheep is an interesting trait not only from a breeding but also from an evolutionary point of view. In most contemporary production sheep breeds, almost all animals are polled (absence of horns), while horns are found mainly in autochthonous breeds. Some sheep breeds, such as the Poll Dorset were specifically bred after the model of the horned basic breed (Dorset Horn), only hornless. Besides sheep breeds fixed either for horns or polledness, there are also those in which one or both sexes have a variable horn status, and even those in which rams are always horned and females are always polled (Table 2, Figure 6). Considering the representatives of the first breed panel of the International Sheep Genome Consortium (ISGC) as a cross-section of the total population (including wild sheep), it can be seen that the majority of the breeds (~39%) are completely hornless, in ~28% of the breeds, the horn status is fixed in one sex and variable in the other, completely variable horn status is present in ~13% of these breeds, whereby strictly sex-specific horns account for 12%, and in only 8% of the breeds studied, all individuals are horned [12,66]. Therefore, the inheritance of horns in sheep varies according to breed and is more complicated than in goat and cattle. Initially, a model with three alleles was proposed, as horn growth in sheep was thought to be controlled by a single autosomal locus [37,67]. The mode of inheritance differs between sexes and it was proposed that the allele that results in horns is dominant in males and recessive in females [68]. 

**Figure 7:**
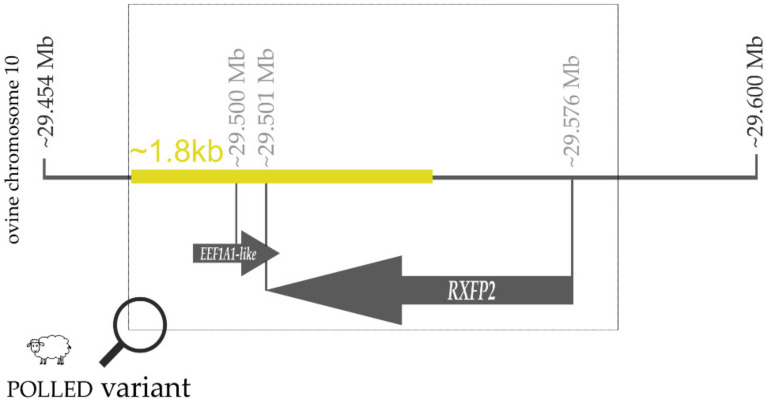
Schematic representation of the polledness-associated insertion variant in sheep in the 3′UTR region of the RXFP2 gene on chromosome 10. The coordinates refer to the current reference genome ARS-UI_Ramb_v2.0 of the sheep. Note that this variant is not associated with polledness in breeds with a sex-linked or variable horn status.

Recent results in Merino sheep confirmed the influence of sex on horn status in this breed [69]. Independent genomic analyses pointed towards a single autosomal locus on chromosome 10 harboring the variant that causes polledness in sheep [68,70]. Pickering et al. (2010) identified a 1.8-kb insertion in the 3’-untranslated region of the ovine *RXFP2* gene located in this region (Figure 7), which was also independently described by Wiedemar and Drögemüller (2015) and present in polled sheep only [49,71]. For Merino sheep, two highly significant associated SNP markers (OAR10_29546872.1, OAR10_29458450) were found near the 1.8-kb insertion, but they still cannot fully explain the genetic diversity regarding the presence/absence of horns in this breed. However, Duijvesteijn et al. (2018) stated that if genotype GG at the marker OAR10_29458450 or TT at marker OAR10_29546872.1 is taken into account, a reliable prediction of non-horned male Merino sheep is possible. 

However, it was recognized that this polledness-associated 1.8-kb insertion variant, which adds a potential antisense RNA sequence of *eukaryotic translation elongation factor 1 alpha 1* (*EEF1A1*) to the 3′-end of *RXFP2* transcripts, does not segregate perfectly with the polled phenotype in sheep breeds with variable or sex-linked horn status [72]. Therefore, it was concluded that the observed variant cannot be the only cause of polledness in sheep. Nevertheless, as far as we know, no other polled-associated alleles have been discovered in sheep so far (OMIA 000483-9940).

The rams of the African Dorper sheep breed can have normal horns or scurs or be hornless, whereas female Dorper ewes are scurred or polled. Interestingly, this breed is fixed for the *RXFP2*-related 1.8-kb insertion [72]. Publicly available short-read whole-genome sequencing data from nine male Dorper sheep with known different horn statuses (four horned, three scurred, and two polled, Table S1) were explored using the current sheep reference genome assembly (ARS-UI_Ramb_v2.0) to search for possible additional *RXFP2*-associated alleles. A visual inspection of the region of the *RXFP2* gene using the integrated genome viewer (IGV) [73] revealed no evidence of novel variants (data not shown). Nevertheless, it might be helpful to use other techniques, such as long-read sequencing, to study the region of interest with a focus on more complex and structural variants, as this was also the successful approach to uncovering the genetic features of the complex PIS-associated variant in goats [56].

The localization of the homologous sequences of the caprine PIS region ruled out the possibility that intersexuality in sheep has the same or similar underlying genetic causes as in goats [74]. However, there is also no evidence of a comparable relationship between polledness and intersexuality in sheep. A small test series (14 breeds, 26 individuals, Table S2) in our laboratory has shown that the application of the published PCR-based detection of the caprine PIS-associated variant using genomic DNA from sheep revealed only the presence of the wild type allele (data not published), supporting the results of Li et al. (2020).

In addition to the mere presence or absence of horns (including scurs), the expression of horn shape and size in sheep also varies. In studies of the isolated Soay sheep population on St. Kilda, the locus for these continuous traits could also be located in the *RXFP2* gene region on chromosome 10 [31]. This suggests that other or even all variants affecting horn growth in sheep may be related to the gene region around *RXFP2* [15,31]. Whole-genome sequencing of Chinese sheep breeds found eight *RXFP2*-related markers that segregate, at least partly, with horn morphology, in terms of length and shape [32].

## 5. Recent Developments in Genetic Engineering Offer New Possibilities for Breeding Hornless Ruminants—First Examples and Current Legal Limits

Even though European law strictly limits the use of new genetic engineering since the landmark ruling of July 2018 [75], the so-called “genetic scissors” techniques, such as transcription activator-like effector nucleases (TALEN) and clustered regularly interspaced short palindromic repeats (CRISPR [76]) are still seen as a great opportunity in agricultural science. The principle behind the application of these nucleases is to trigger a DNA double-strand break at a previously defined location in the genome, which is repaired in the cells in one of two possible ways. Firstly, there is the repair mechanism of non-homologous end-joining in which resulting fragments are ligated without an external template, enabling gene knock-outs. Secondly, there is the homology-directed repair, in which a predesigned template is used as a pattern for ligating the fragments. This makes this method suitable for gene knock-in or the replacement of a specific sequence in general [77,78,79].

As one of the first successful applications in domestic animals, the “*Celtic*” polled allele (*PC*) of cattle was independently integrated into the genomes of horned cattle [80,81,82]. Moreover, it was shown that all heterozygous progeny of dairy bulls that became homozygous for *PC* after genome editing expressed the polled trait as expected. At the same time, other intended changes in the genome sequence were not detectable [83]. 

Since polledness in cattle is apparently not associated with any harm, it is possible to spread the desired trait through conventional breeding within a few generations in previously horned populations without any negative effects. However, to give genome editing an edge, some argue that the population of hornless breeding bulls with reliable and good breeding values (comparable to horned counterparts) is too small to avoid inbreeding while supporting the introgression of the polled trait. For this reason, it takes much longer to increase the frequency of polledness in a population using conventional breeding strategies than with genetic engineering [84,85,86]. From a basic science perspective, since it appears that more than one variant affecting different genes is causing polledness, genome editing might give the opportunity of demonstrating which of the variants is crucial for the absence of horns. Alternatively, confirmation of the interaction of several variants as a cause for polledness would be possible through it, as recently done for the PC variant in cattle [87].

These techniques have also been successfully applied to sheep and goats when dealing with issues other than polledness [88,89]. For these two species in particular, and the unique features of the inheritance of polledness in them, the use of genome editing would offer new approaches and opportunities to establish the desired trait in the respective population. In goats in particular, it might be helpful to find a way to introduce inherited polledness into the population without the associated intersexuality. One possibility would be an attempt to insert one of the bovine variants for polledness, preferably the less complex *PC* variant, into the goat genome. So far, no such attempts have been published.

However, in addition to strict legislation in Europe, there are also reservations among the public about genetically modified animals for consumption. Both ethical concerns and risk–benefit assessments of genetic engineering in food production explain the critical attitude of consumers. In general, it can be summarized that Europeans are more critical of the topic than American and Asian consumers [90,91,92]. Finally, there are also critical voices that claim, on the basis of these supposed examples of success with hornless cattle, that this is the wrong way to solve the problem, as it is merely a "technological solution" to a complex social problem [93].

## 6. Conclusions

Although the presence or absence of horns is a trait as old as livestock, it is a topic that never loses its relevance. The study of horn phenotypes in ruminants confirms the still underestimated role of domestic animals as unique models for biomedical research due to their long history (thousands of years) of strong phenotypic selection. The three known hornless loci in cattle, goat, and sheep each affect different genes, although the resulting phenotypes with the absence of horn growth are more or less identical. This confirms the assumed heterogeneity and complexity that determines the development of these organs, which are unique in the animal kingdom. Nevertheless, the underlying genetic mechanisms, especially in sheep, remain largely unknown, highlighting the need for further research in this field. A challenge will be to clarify the implementation of intermediate phenotypes, such as scurs and sex-linked factors. In addition, the underlying mechanisms in all three ruminant species still need to be investigated. There is no doubt that breeding for polledness is a sensible and permanent alternative to surgical dehorning in order to take animal welfare into account and offer an animal-friendly alternative [27,84].

The new possibilities offered by genome editing techniques could serve as a tool to spread this trait faster than through conventional breeding, especially in cattle. In goats, it may be possible to specifically modify the corresponding genomic regions that are altered in cattle or sheep to avoid the negative association of the naturally occurring hornless variant with intersexuality.

## Figures and Tables

**Table 1:** Impact of the caprine *POLLED* allele on horn status and the fertility of female (XX) and male (XY) goats.

	Genotype at the *POLLED* Locus		
Genetic Sex	pp (Homozygous; Wild Type)	Pp (Heterozygous)	PP (Homozygous)
XX—female	horned/fertile	polled/fertile	polled/infertile—intersex (normal outer phenotype or “pseudo-buck” to variable degrees
XY—male	horned/fertile	polled/fertile	polled/fertility unclear

**Table 2:** List of sheep breeds (*Ovis aries*), including the European mouflon (*Ovis musimon*), showing different horn phenotypes between sexes. Individuals of the respective sex and breed are either horned, polled, or the horn status is variable (there are polled, horned, and scurred individuals).

Horn Status Group	Breed	Species	Horn Status Females	Horn Status Males
Completely polled	Barbados Blackbelly Sheep *	*Ovis aries*	Polled	Polled
	Bentheimer Charollais	*Ovis aries* *Ovis aries*	Polled Polled	Polled Polled
	Coburger East Friesian Milk Sheep * German Black-headed Mutton German Brown Mountain German White Mountain Ile de France Kerry Hill Sheep * Lacaune Sheep * Merinoland Sheep * Poll Dorset Roughwool Pomeranian Sheep * Rhone Sheep Suffolk Texel Sheep *	*Ovis aries* *Ovis aries* *Ovis aries* *Ovis aries* *Ovis aries* *Ovis aries* *Ovis aries* *Ovis aries* *Ovis aries* *Ovis aries* *Ovis aries* *Ovis aries* *Ovis aries* *Ovis aries*	Polled Polled Polled Polled Polled Polled Polled Polled Polled Polled Polled Polled Polled Polled	Polled Polled Polled Polled Polled Polled Polled Polled Polled Polled Polled Polled Polled Polled
Completely horned	Grey Horned Heath * Scottish Blackface Sheep * Valais Blacknose Sheep Mouflon *	*Ovis aries* *Ovis aries* *Ovis aries* *Ovis musimon*	Horned Horned Horned Horned	Horned Horned Horned Horned
Variable in both sexes	African Dorper Sheep * Alpines Steinschaf Icelandic Sheep Krainer Steinschaf * Soay Sheep *	*Ovis aries* *Ovis aries* *Ovis aries* *Ovis aries* *Ovis aries*	Variable Variable Variable Variable Variable	Variable Variable Variable Variable Variable
Strictly sex-linked	Ethiopian Menz Cameroon Sheep *	*Ovis aries* *Ovis aries*	Polled Polled	Horned Horned
	Rambouillet	*Ovis aries*	Polled	Horned
Males horned, females variable	Walachian Sheep	*Ovis aries*	Variable	Horned
	Ouessant Sheep *	*Ovis aries*	Mostly polled	Horned

* Note that individuals of the marked breeds were genotyped as wild type for the goat PIS-related complex variant.

## Data Availability

Genome sequencing data were deposited in the European Nucleotide Archive (ENA, http://www.ebi.ac.uk/ena accessed on 20 February 2022).

## Supplementary Materials

The following supporting information can be downloaded at https://www.mdpi.com/article/10.3390/genes13050832/s1, Table S1: Whole-genome sequencing data from Dorper sheep.; Table S2: Sheep genotyped with genetic testing for polled intersex syndrome in goats.

## References

[1] Allais-Bonnet A., Hintermann A., Deloche M.-C., Cornette R., Bardou P., Naval-Sanchez M., Pinton A., Haruda A., Grohs C., Zakany J. (2021). Analysis of Polycerate Mutants Reveals the Evolutionary Co-option of HOXD1 for Horn Patterning in Bovidae. Mol. Biol. Evol..

[2] Wang Y., Zhang C., Wang N., Li Z., Heller R., Liu R., Zhao Y., Han J., Pan X., Zheng Z. (2019). Genetic basis of ruminant headgear and rapid antler regeneration. Science.

[3] Geist V. (1966). The Evolution of Horn-Like Organs. Behaviour.

[4] Lincoln G.A., Short R.V., Bulaban E. (1994). Teeth, horns and antlers: The weapons of sex. The Difference between the Sexes.

[5] Stankowich T., Caro T. (2009). Evolution of weaponry in female bovids. Proc. Biol. Sci..

[6] Estes R.D. (1991). The significance of horns and other male secondary sexual characters in female bovids. Appl. Anim. Behav. Sci..

[7] Maity P., Tekalur S.A. (2011). Finite element analysis of ramming in Ovis canadensis. J. Biomech. Eng..

[8] Preston B.T., Stevenson I.R., Pemberton J.M., Coltman D.W., Wilson K. (2003). Overt and covert competition in a promiscuous mammal: The importance of weaponry and testes size to male reproductive success. Proc. Biol. Sci..

[9] Robinson M.R., Kruuk L. (2007). Function of weaponry in females: The use of horns in intrasexual competition for resources in female Soay sheep. Biol. Lett..

[10] Picard K., Thomas D.W., Festa-Bianchiet M., Belleville F., Laneville A. (1999). Differences in thermal conductivity of tropical and temperate bovid horns. Ecoscience.

[11] Parés-Casanova P., Caballero M. (2014). Possible tendency of polled cattle towards larger ears. Revista Colombiana de Ciencias Pecuarias.

[12] Porter V., Alderson L., Hall S., Sponenberg D.P. (2016). Masons World Encyclopedia of Livestock Breeds and Breeding: 2 Volume Pack.

[13] Stookey J.M., Goonewardene L.A. (1996). A comparison of production traits and welfare implications between horned and polled beef bulls. Can. J. Anim. Sci..

[14] Scheper C., Emmerling R., Götz K.-U., König S. (2021). A variance component estimation approach to infer associations between Mendelian polledness and quantitative production and female fertility traits in German Simmental cattle. Genet. Sel. Evol..

[15] Johnston S.E., Gratten J., Berenos C., Pilkington J.G., Clutton-Brock T.H., Pemberton J.M., Slate J. (2013). Life history trade-offs at a single locus maintain sexually selected genetic variation. Nature.

[16] Goldblum D., Frueh B.E., Koerner F. (1999). Eye injuries caused by cow horns. Retina.

[17] Knierim U., Irrgang N., Roth B.A. (2015). To be or not to be horned—Consequences in cattle. Livest. Sci..

[18] Braun U., Gerspach C., Stettler M., Grob D., Sydler T. (2016). Rumen perforation caused by horn injury in two cows. Acta Vet. Scand..

[19] Youngers M.E., Thomson D.U., Schwandt E.F., Simroth J.C., Bartle S.J., Siemens M.G., Reinhardt C.D. (2017). Case Study: Prevalence of horns and bruising in feedlot cattle at slaughter. Prof. Anim. Sci..

[20] Menke C., Waiblinger S., Fölsch D.W., Wiepkema P.R. (1999). Social behaviour and injuries of horned cows in loose housing systems. Anim. Welf..

[21] Waiblinger S., Schmied-Wagner C., Nordmann E., Mersmann D., Szabo S., Graml C., von Hof J., Maschat K., Grubmüller T., Winckler C. (2010). Haltung von Behornten und Unbehornten Milchziegen in Großgruppen.

[22] Cozzi G., Gottardo F., Brscic M., Contiero B., Irrgang N., Knierim U., Pentelescu O., Windig J.J., Mirabito L., Kling Eveillard F. (2015). Dehorning of cattle in the EU Member States: A quantitative survey of the current practices. Livest. Sci..

[23] Hempstead M.N., Lindquist T.M., Shearer J.K., Shearer L.C., Plummer P.J. (2021). Health and Welfare Survey of 30 Dairy Goat Farms in the Midwestern United States. Animals.

[24] (1998). Council Directive 98/59/EC concering the protection of animals kept for farming purpose: 98/58/EC, CELEX-EUR. CELEX-EUR Off. J. L 221.

[25] Cozzi G., Prevedello P., Boukha A., Winckler C., Knierim U., Pentelescu O., Windig J.J., Mirabito L., Kling Eveillard F., Dockes A.C. (2009). Alternatives to Castration and Dehorning. Report on Dehorning Practices across EU Member States.: SP2: Alternatives to Dehorning: To Develop and Promote Alternatives to the Dehorning of Cattle. WP2.1: State of the Art of Dehorning in the EU Member States. ALCASDE; SANCO/2008/D5/018). https://ec.europa.eu/food/system/files/2016-10/aw_prac_farm_pigs_cast-alt_research_alcasade_final-report.pdf.

[26] (2006). Tierschutzgesetz: TSchG. https://www.gesetze-im-internet.de/tierschg/BJNR012770972.html.

[27] Prayaga K.C. (2007). Genetic options to replace dehorning in beef cattle—A review. Aust. J. Agric. Res..

[28] (2008). Commission Regulation (EC). No 889/2008 of 5 September 2008 Laying Down Detailed Rules for the Implementation of Council Regulation (EC) No 834/2007 on Organic Production and Labelling of Organic Products with Regard to Organic Production, Labelling and Control. http://data.europa.eu/eli/reg/2008/889/oj.

[29] Still Brooks K.M., Hempstead M.N., Anderson J.L., Parsons R.L., Sutherland M.A., Plummer P.J., Millman S.T. (2021). Characterization of Efficacy and Animal Safety across Four Caprine Disbudding Methodologies. Animals.

[30] Castle W.E. (1940). Genetics of horns in sheep. J. Hered..

[31] Johnston S.E., Beraldi D., McRae A.F., Pemberton J.M., Slate J. (2010). Horn type and horn length genes map to the same chromosomal region in Soay sheep. Heredity.

[32] Pan Z., Li S., Liu Q., Wang Z., Zhou Z., Di R., Miao B., Hu W., Wang X., Hu X. (2018). Whole-genome sequences of 89 Chinese sheep suggest role of RXFP2 in the development of unique horn phenotype as response to semi-feralization. Gigascience.

[33] Clutton-Brock T.H., Wilson K., Stevenson I.R. (1997). Density-dependent selection on horn phenotype in Soay sheep. Philos. Trans. R. Soc. Lond. B Biol. Sci..

[34] Schafberg R., Swalve H.H. (2015). The history of breeding for polled cattle. Livest. Sci..

[35] Warwick B.L., Dunkle P.B. (1939). Inheritance of horns in sheep: Triple Alleles in a Dorset-Rambouillet Cross. J. Hered..

[36] Gehrke L.J., Capitan A., Scheper C., König S., Upadhyay M., Heidrich K., Russ I., Seichter D., Tetens J., Medugorac I. (2020). Are scurs in heterozygous polled (Pp) cattle a complex quantitative trait?. Genet. Sel. Evol..

[37] Clutton-Brock T.H., Pemberton J.M. (2004). Soay Sheep: Population Dynamics and Selection on St. Kilda.

[38] Wiener D.J., Wiedemar N., Welle M.M., Drögemüller C. (2015). Novel Features of the Prenatal Horn Bud Development in Cattle (*Bos taurus*). PLoS ONE.

[39] Egyptian Museum Relief of a Man Milking a Cow; Carving on the Sarcophagus of Queen Kawit; Deir el-Bahari, West Thebes, ~2061–2010 B.C. http://www.globalegyptianmuseum.org/record.aspx?id=15277.

[40] Aldersey J.E., Sonstegard T.S., Williams J.L., Bottema C.D.K. (2020). Understanding the effects of the bovine POLLED variants. Anim. Genet..

[41] Georges M., Drinkwater R., King T., Mishra A., Moore S.S., Nielsen D., Sargeant L.S., Sorensen A., Steele M.R., Zhao X. (1993). Microsatellite mapping of a gene affecting horn development in *Bos taurus*. Nat. Genet..

[42] White W.T., Ibsen H.L. (1936). Horn inheritance in Galloway-Holstein cattle crosses. J. Genet..

[43] Bateson W., Saunders E.R. (1902). The facts of heredity in the light of Mendel’s discovery. Rep. Evol. Comm. R. Soc..

[44] Medugorac I., Seichter D., Graf A., Russ I., Blum H., Göpel K.H., Rothammer S., Förster M., Krebs S. (2012). Bovine polledness--an autosomal dominant trait with allelic heterogeneity. PLoS ONE.

[45] Rothammer S., Capitan A., Mullaart E., Seichter D., Russ I., Medugorac I. (2014). The 80-kb DNA duplication on BTA1 is the only remaining candidate mutation for the polled phenotype of Friesian origin. Genet. Sel. Evol..

[46] Allais-Bonnet A., Grohs C., Medugorac I., Krebs S., Djari A., Graf A., Fritz S., Seichter D., Baur A., Russ I. (2013). Novel insights into the bovine polled phenotype and horn ontogenesis in Bovidae. PLoS ONE.

[47] Mariasegaram M., Reverter A., Barris W., Lehnert S.A., Dalrymple B., Prayaga K. (2010). Transcription profiling provides insights into gene pathways involved in horn and scurs development in cattle. BMC Genom..

[48] Wiedemar N., Tetens J., Jagannathan V., Menoud A., Neuenschwander S., Bruggmann R., Thaller G., Drögemüller C. (2014). Independent polled mutations leading to complex gene expression differences in cattle. PLoS ONE.

[49] Wiedemar N., Drögemüller C. (2015). A 1.8-kb insertion in the 3′-UTR of RXFP2 is associated with polledness in sheep. Anim. Genet..

[50] Boulanger L., Pannetier M., Gall L., Allais-Bonnet A., Elzaiat M., Le Bourhis D., Daniel N., Richard C., Cotinot C., Ghyselinck N.B. (2014). FOXL2 is a female sex-determining gene in the goat. Curr. Biol..

[51] Medugorac I., Graf A., Grohs C., Rothammer S., Zagdsuren Y., Gladyr E., Zinovieva N., Barbieri J., Seichter D., Russ I. (2017). Whole-genome analysis of introgressive hybridization and characterization of the bovine legacy of Mongolian yaks. Nat. Genet..

[52] Utsunomiya Y.T., Torrecilha R.B.P., Milanesi M., Paulan S.d.C., Utsunomiya A.T.H., Garcia J.F. (2019). Hornless Nellore cattle (Bos indicus) carrying a novel 110 kbp duplication variant of the polled locus. Anim. Genet..

[53] Randhawa I.A.S., Burns B.M., McGowan M.R., Porto-Neto L.R., Hayes B.J., Ferretti R., Schutt K.M., Lyons R.E. (2020). Optimized Genetic Testing for Polledness in Multiple Breeds of Cattle. G3.

[54] Capitan A., Grohs C., Weiss B., Rossignol M.-N., Reversé P., Eggen A. (2011). A newly described bovine type 2 scurs syndrome segregates with a frame-shift mutation in TWIST1. PLoS ONE.

[55] Pailhoux E., Vigier B., Chaffaux S., Servel N., Taourit S., Furet J.P., Fellous M., Grosclaude F., Cribiu E.P., Cotinot C. (2001). A 11.7-kb deletion triggers intersexuality and polledness in goats. Nat. Genet..

[56] Simon R., Lischer H.E.L., Pieńkowska-Schelling A., Keller I., Häfliger I.M., Letko A., Schelling C., Lühken G., Drögemüller C. (2020). New genomic features of the polled intersex syndrome variant in goats unraveled by long-read whole-genome sequencing. Anim. Genet..

[57] Guo J., Jiang R., Mao A., Liu G.E., Zhan S., Li L., Zhong T., Wang L., Cao J., Chen Y. (2021). Genome-wide association study reveals 14 new SNPs and confirms two structural variants highly associated with the horned/polled phenotype in goats. BMC Genom..

[58] Pannetier M., Elzaiat M., Thépot D., Pailhoux E. (2012). Telling the story of XX sex reversal in the goat: Highlighting the sex-crossroad in domestic mammals. Sex Dev..

[59] Soller M., Padeh B., Wysoki M., Ayalon N. (1969). Cytogenetics of Saanen goats showing abnormal development of the reproductive tract associated with the dominant gene for polledness. Cytogenetics.

[60] Asdell S.A. (1944). The genetic sex of intersexual goats and a probable linkage with the gene for hornlessness. Science.

[61] Szatkowska I., Zabarski D., Proskura W.S., Tabor S. (2014). Polledness intersex syndrome in goats–molecular and histological aspects. Turk. J. Vet. Anim. Sci..

[62] Yadav B.R., Singh C., Kumar P., Tomer O.S., Yadav J.S. (1993). Morphological, anatomical and cytogenetical investigations in sexually anomalous goats. Small Rumin. Res..

[63] Pannetier M., Renault L., Jolivet G., Cotinot C., Pailhoux E. (2005). Ovarian-specific expression of a new gene regulated by the goat PIS region and transcribed by a FOXL2 bidirectional promoter. Genomics.

[64] Zlotorynski E. (2016). Genome organization: Add a TAD of duplication. Nat. Rev. Mol. Cell Biol..

[65] Franke M., Ibrahim D.M., Andrey G., Schwarzer W., Heinrich V., Schöpflin R., Kraft K., Kempfer R., Jerković I., Chan W.-L. (2016). Formation of new chromatin domains determines pathogenicity of genomic duplications. Nature.

[66] Archibald A.L., Cockett N.E., Dalrymple B.P., Faraut T., Kijas J.W., Maddox J.F., McEwan J.C., Hutton Oddy V., Raadsma H.W., Wade C. (2010). The sheep genome reference sequence: A work in progress. Anim. Genet..

[67] Dolling C. (1961). Hornedness and polledness in sheep.: IV. Triple alleles affecting horn growth in the Merino. Aust. J. Agric. Res..

[68] Johnston S.E., McEwan J.C., Pickering N.K., Kijas J.W., Beraldi D., Pilkington J.G., Pemberton J.M., Slate J. (2011). Genome-wide association mapping identifies the genetic basis of discrete and quantitative variation in sexual weaponry in a wild sheep population. Mol. Ecol..

[69] Duijvesteijn N., Bolormaa S., Daetwyler H.D., van der Werf J.H.J. (2018). Genomic prediction of the polled and horned phenotypes in Merino sheep. Genet. Sel. Evol..

[70] Dominik S., Henshall J.M., Hayes B.J. (2012). A single nucleotide polymorphism on chromosome 10 is highly predictive for the polled phenotype in Australian Merino sheep. Anim. Genet..

[71] Pickering N.K., Johnson P.L., Auvray B., Dodds K.G., McEwan J.C. (2009). Mapping the horns locus in sheep. Proc. Assoc. Advmt. Anim. Breed. Genet.

[72] Lühken G., Krebs S., Rothammer S., Küpper J., Mioč B., Russ I., Medugorac I. (2016). The 1.78-kb insertion in the 3’-untranslated region of RXFP2 does not segregate with horn status in sheep breeds with variable horn status. Genet. Sel. Evol..

[73] Robinson J.T., Thorvaldsdóttir H., Winckler W., Guttman M., Lander E.S., Getz G., Mesirov J.P. (2011). Integrative genomics viewer. Nat. Biotechnol..

[74] Li J., Xu H., Liu X., Xu H., Cai Y., Lan X. (2020). Insight into the Possible Formation Mechanism of the Intersex Phenotype of Lanzhou Fat-Tailed Sheep Using Whole-Genome Resequencing. Animals.

[75] EuGH (2018). “Vorlage zur Vorabentscheidung–Absichtliche Freisetzung genetisch veränderter Organismen in die Umwelt–Mutagenese–Richtlinie 2001/18/EG–Art. 2 und 3–Anhänge I A und I B–Begriff ‚genetisch veränderter Organismus‘–Herkömmlich angewandte und als sicher geltende Verfahren/Methoden zur genetischen Veränderung–Neue Verfahren/Methoden der Mutagenese–Risiken für die menschliche Gesundheit und die Umwelt–Ermessen der Mitgliedstaaten bei der Umsetzung der Richtlinie–Richtlinie 2002/53/EG–Gemeinsamer Sortenkatalog für landwirtschaftliche Pflanzenarten–Herbizidtolerante Pflanzensorten–Art. 4–Zulassung durch Mutagenese gewonnener genetisch veränderter Sorten zum gemeinsamen Sortenkatalog–Anforderung zum Schutz der menschlichen Gesundheit und der Umwelt–Befreiung”. https://curia.europa.eu/juris/document/document.jsf?text=&docid=204387&pageIndex=0&doclang=DE&mode=req&dir=&occ=first&part=1.

[76] Jinek M., Chylinski K., Fonfara I., Hauer M., Doudna J.A., Charpentier E. (2012). A programmable dual-RNA-guided DNA endonuclease in adaptive bacterial immunity. Science.

[77] Ruan J., Xu J., Chen-Tsai R.Y., Li K. (2017). Genome editing in livestock: Are we ready for a revolution in animal breeding industry?. Transgenic Res..

[78] Van Eenennaam A.L. (2018). The contribution of transgenic and genome-edited animals to agricultural and industrial applications. Rev. Sci. Tech..

[79] Doudna J.A., Charpentier E. (2014). Genome editing. The new frontier of genome engineering with CRISPR-Cas9. Science.

[80] Carlson D.F., Lancto C.A., Zang B., Kim E.-S., Walton M., Oldeschulte D., Seabury C., Sonstegard T.S., Fahrenkrug S.C. (2016). Production of hornless dairy cattle from genome-edited cell lines. Nat. Biotechnol..

[81] Schuster F., Frenzel A., Petersen B., Lucas-Hahn A., Boch J., Nieman H. (2018). Generierung eines Hornlos-Phänotyps in Holstein-Friesian und Braunvieh Bullen durch Einsatz von DNA-Nukleasen. Aus der Arbeit der Forschungsstätten für Tierwissenschaften, Kurzfassungen, Proceedings of the Vortragstagung der GDfZ und GfT, Bonn, Germany, 12–13 October 2018.

[82] Schuster F., Aldag P., Frenzel A., Hadeler K.-G., Lucas-Hahn A., Niemann H., Petersen B. (2020). CRISPR/Cas12a mediated knock-in of the Polled Celtic variant to produce a polled genotype in dairy cattle. Sci. Rep..

[83] Young A.E., Mansour T.A., McNabb B.R., Owen J.R., Trott J.F., Brown C.T., van Eenennaam A.L. (2020). Genomic and phenotypic analyses of six offspring of a genome-edited hornless bull. Nat. Biotechnol..

[84] Windig J.J., Hoving-Bolink R.A., Veerkamp R.F. (2015). Breeding for polledness in Holstein cattle. Livest. Sci..

[85] Mueller M.L., Cole J.B., Sonstegard T.S., van Eenennaam A.L. (2019). Comparison of gene editing versus conventional breeding to introgress the POLLED allele into the US dairy cattle population. J. Dairy Sci..

[86] Mueller M.L., Cole J.B., Connors N.K., Johnston D.J., Randhawa I.A.S., van Eenennaam A.L. (2021). Comparison of Gene Editing Versus Conventional Breeding to Introgress the POLLED Allele Into the Tropically Adapted Australian Beef Cattle Population. Front. Genet..

[87] Hennig S.L., Owen J.R., Lin J.C., McNabb B.R., van Eenennaam A.L., Murray J.D. (2022). A deletion at the polled PC locus alone is not sufficient to cause a polled phenotype in cattle. Sci. Rep..

[88] Proudfoot C., Carlson D.F., Huddart R., Long C.R., Pryor J.H., King T.J., Lillico S.G., Mileham A.J., McLaren D.G., Whitelaw C. (2015). Genome edited sheep and cattle. Transgenic Res..

[89] Wang X., Yu H., Lei A., Zhou J., Zeng W., Zhu H., Dong Z., Niu Y., Shi B., Cai B. (2015). Generation of gene-modified goats targeting MSTN and FGF5 via zygote injection of CRISPR/Cas9 system. Sci. Rep..

[90] Frewer L.J., Coles D., Houdebine L.-M., Kleter G.A. (2013). Attitudes towards genetically modified animals in food production. Br. Food J..

[91] Frewer L.J., van der Lans I.A., Fischer A.R., Reinders M.J., Menozzi D., Zhang X., van den Berg I., Zimmermann K.L. (2013). Public perceptions of agri-food applications of genetic modification–A systematic review and meta-analysis. Trends Food Sci. Technol..

[92] Canavari M., Nayga R.M. (2009). On consumers’ willingness to purchase nutritionally enhanced genetically modified food. Appl. Econ..

[93] Devolder K. (2021). Genome Editing in Livestock, Complicity, and the Technological Fix Objection. J. Agric. Environ. Ethics.

